# Reference values for diaphragm electrical activity (Edi) in newborn infants

**DOI:** 10.1186/s12887-022-03619-1

**Published:** 2022-09-23

**Authors:** Varappriyangga Gurumahan, Sriganesh Thavalingam, Tim Schindler, John Smyth, Kei Lui, Srinivas Bolisetty

**Affiliations:** 1 School of Women’s and Children’s Health, University of New South Wales, Sydney, Australia; 2 Department of Newborn Care, Royal Hospital for Women, Newborn Care, Royal Hospital for Women, Barker St, Randwick, Sydney, NSW 2031 Australia

**Keywords:** Intensive care units, Neonatal, Non-invasive ventilation, Diaphragm

## Abstract

**Background:**

Neurally adjusted ventilatory assist is an emerging mode of respiratory support that uses the electrical activity of the diaphragm (Edi) to provide synchronised inspiratory pressure support, proportional to an infant’s changing inspiratory effort. Data on Edi reference values for neonates are limited. The objective of this study was to establish reference Edi values for preterm and term neonates who are not receiving respiratory support.

**Methods:**

This was a prospective observational study of newborn infants breathing spontaneously in room air. The Edi waveform was monitored by a specialised naso/orogastric feeding tube with embedded electrodes positioned at the level of the diaphragm. Edi minimums and peaks were recorded continuously for 4 h without changes to routine clinical handling.

**Results:**

Twenty-four newborn infants (16 preterm [< 37 weeks’ gestation]; 8 term) were studied. All infants were breathing comfortably in room air at the time of study. Edi data were successfully captured in all infants. The mean (±SD) Edi minimum was 3.02 (±0.94) μV and the mean Edi peak was 10.13 (±3.50) μV. In preterm infants the mean (±SD) Edi minimum was 3.05 (±0.91) μV and the mean Edi peak was 9.36 (±2.13) μV. In term infants the mean (±SD) Edi minimum was 2.97 (±1.05) μV and the mean Edi peak was 11.66 (±5.14) μV.

**Conclusion:**

Reference Edi values were established for both preterm and term neonates. These values can be used as a guide when monitoring breathing support and when using diaphragm-triggered modes of respiratory support in newborn infants

**Supplementary Information:**

The online version contains supplementary material available at 10.1186/s12887-022-03619-1.

## Background

The initiation of a spontaneous breath by the respiratory centre of the brain results in the electrical excitation of the diaphragm via the phrenic nerve and subsequent diaphragmatic contraction to facilitate an inflow of air. Recently, new technology has become available that can measure diaphragmatic excitation and associated electrical activity of the diaphragm (Edi) [1]. This is achieved using an Edi catheter, which is a specialised naso/orogastric tube that has electrodes embedded at the level of the gastro-oesophageal junction. The catheter assesses neural respiratory control by measuring the total action potentials of the motor units in the diaphragmatic crura [1], representative of phrenic nerve activity and global diaphragm activation [2]. The Edi is presented as a waveform with a cyclic pattern that has distinctive peak and minimum values. There are two key measures from this Edi waveform: (1) Edi minimum (the end expiratory Edi value [just prior to the subsequent inspiration]); and (2) Edi peak (the amplitude of electrical activity associated with inspiratory effort) [3].

These Edi waveforms can be filtered, processed and relayed to a ventilator, which can then provide synchronised inspiratory pressure support. The ventilator provides support that is proportional to inspiratory effort, based on the difference between the Edi peak and Edi minimum. This modality, referred to as neurally adjusted ventilatory assist (NAVA), is an emerging mode of respiratory support that can be utilised either non-invasively via nasal prongs or mask, or invasively [4]. Over 100 clinical and experimental studies have demonstrated the benefit of neural monitoring and ventilator assist control compared to conventional modes of ventilation, during both non-invasive and invasive ventilation. These studies have shown that NAVA improves patient-ventilator interaction, offering equivalent or improved physiological outcomes and favourable ventilator parameters (lower peak inspiratory pressures and down-regulation of Edi to limit over-assist) [5, 6].

Although several studies have evaluated the clinical use of NAVA in newborn infants, reference Edi data in healthy neonates have previously only been collected in two studies, involving 20 infants [3, 7]. However, data pertaining to healthy preterm and term infants not requiring respiratory support is limited. Establishing reference values for this population is important for the clinical utility of this technology. Excessive respiratory support can suppress an infant’s natural respiratory drive while under supporting breathing is also detrimental [8]. The potential morbidity and mortality associated with incorrect support is well established [9].

This observational study of preterm and term newborn infants aimed to establish reference Edi values. We also sought to determine how these values vary according to sleeping states, feeding and skin-to-skin care.

## Methods

### Patient selection

Newborn infants born at 29–42 weeks’ gestation who were admitted to the Neonatal Intensive Care Unit (NICU) at the Royal Hospital for Women were opportunistically recruited for this prospective observational study. Inclusion criteria required that neonates were on room air with no current respiratory issues and with a naso/orogastric feeding tube in situ. Exclusion criteria included infants that had been on respiratory support within the last week, congenital lung anomalies, major neurological conditions and brain anomalies, neuromuscular diseases and conditions affecting innervation of the diaphragm.

### Study procedure

An Edi catheter (6 Fr) was inserted when the infant’s conventional naso/orogastric feeding tube was due to be changed as per unit policy. The Edi catheter was positioned at the level of the diaphragm (starting with a measurement of the direct distance from nose to earlobe to xiphisternum [NEX]) and the correct position confirmed by analysis using Servo-n software (Getinge AB Gothenburg, Sweden). The Edi waveform was recorded continuously for the duration of the study. It was adjusted so that in the “catheter positioning screen”, the Edi waveform, superimposed as a pink waveform, was noted to occur on the middle 2 out of 4 retro-cardiac ECG tracing. Data output from the Servo-n included Edi peak, Edi min and respiratory rate. The Servo-n stored the data in 1 min (averaged) increments, which were then extracted and analysed. It is important to note that infants may have had sighs (high Edi peak) or apneoas (no Edi waveform), which would not be represented due to this 1 min averaging.

Infants were observed for a continuous four-hour period, resulting in 240 data points for each variable observed (see example Additional file 1). Heart rate and oxygen saturation (SpO2) were also recorded from telemetry monitors every 15 minutes throughout the study. An observer subjectively recorded sleep, awake and feeding states by direct observation, as well as timing skin-to-skin care (defined as continuous skin-to-skin contact in a vertical position on the parent’s chest). Feeding states for comparison were defined as 1 h pre- and post-prandial. During the study period, normal clinical activity was not interrupted. On completion of the observation period, the Edi catheter was left in situ for use as a regular nasogastric tube. All infants were studied for a single 4 h period only.

### Statistical analyses

Basic descriptive statistics were performed to calculate population means and standard deviations. Paired sample t-tests were used to compare means between sleep states, feeding and skin-to-skin care. Statistical significance was defined as *p* < 0.05.

## Results

### Patient inclusion

A total of 24 infants (16 preterm; 8 term) were enrolled in the study (Table 1). Birth weights ranged from 1270 g to 3490 g, and postnatal age ranged from day 2 to 34. All infants were breathing comfortably in room air at the time of enrolment. Infants were studied for 240 minutes, and all infants completed the study (individual Edi data available Additional file 2). For feeding state comparisons, at least 45 minutes of data was analysed per subject both pre- and post-prandially. Feeding state comparisons could not be conducted for one infant (not feeding at the time of the study).

**Table 1:** Demographic details of study population

Demographic Characteristic	Number (N)
Sex	
Male	11
Female	13
GA at birth	
29^+0^ - 36^+6^ weeks	16
≥ 37^+0^ weeks	8
Median (range)	34+5 (29+5 – 40+6) weeks
Birth Weight	
<1500g	3
1500-2499g	14
≥2500g	7
Median (range)	2070 (1270-3490) g
Birth Weight <10^th^ percentile	6
Age (in days) at time of study	
Median (range)	10 (2-34) days
GA at time of study^a^	
29^+0^ - 36^+6^ weeks	15
≥ 37^+0^ weeks	9
Median (range)	36+1 (31+2 – 41+1) weeks
Weight at time of study	
<1500g	2
1500-2499g	13
≥2500g	9
Median (range)	2153 (1280-3775) g
Maximum respiratory support prior to time of study	
No Respiratory Support	8
CPAP	6
Low Flow Oxygen	1
High Flow Oxygen	2
Mechanical Ventilation	7
Primary Reason for Admission^b^	
Transient tachypnoea of the newborn	4
Jaundice with phototherapy	1
Surgical review^c^	2
Prematurity	11
Low Birth Weight	2
Neonatal abstinence syndrome	1
Risk of Sepsis	2
Hypoglycaemia	1

^a^One preterm infant had reached term corrected age (exact gestation: 39+3 weeks) at the time of enrolment but was included in the preterm group for analyses

^b^At the time of enrolment, all subjects were breathing comfortably in room air and had no respiratory compromise associated with their primary reason for admission

^c^Both neonates required gastrointestinal surgery (Hirschsprung’s disease; colonic atresia)

Average Edi peak, Edi min, respiratory rate, heart rate and oxygen saturation across all 24 infants are presented in Table 2. Heart rate, respiratory rate and oxygen saturations were all within the normal range for gestational age. The mean (±SD) Edi minimum was 3.02 (±0.94) μV and the mean (±SD) Edi peak was 10.13 (±3.50) μV. Averaged data for the preterm and term infants are also presented. There was no significant difference in Edi minimum (*p* = 0.844) or Edi peak (*p* = 0.259) between preterm and term infants. Based on data from the entire cohort, the reference ranges (defined as mean ± 2 SD) for Edi minimum and Edi peak would be 1–5 μV and 3–17 μV, respectively.

**Table 2:** Edi Min, Edi Peak, Edi Swing, Respiratory Rate, Heart Rate and Oxygen Saturation amongst Population Groups^a,b,c,d^

	N	Edi Min (μV)	Edi Peak (μV)	Edi Swing (μV)	Resp Rate (breaths/min)	Heart Rate (beats/min)	SaO2 (%)
Total	24	3.02 ± 0.94 (CV 0.31)	10.13 ± 3.50 (CV 0.35)	7.10 ± 3.03 (CV 0.43)	47.93 ± 5.43	152.30 ± 12.78	96.00 ± 2.51
Preterm	16	3.05 ± 0.91 (CV 0.30)	9.36 ± 2.13 (CV 0.23)	6.31 ± 1.88 (CV 0.30)	48.47 ± 5.78	145.25 ± 43.12	96.80 ± 1.14
Term	8	2.97 ± 1.05 (CV 0.35)	11.66 ± 5.14 (CV 0.44)	8.69 ± 4.27 (CV 0.49)	39.75 ± 20.17	146.24 ± 9.47	94.50 3.64

^a^Data is presented as mean ± standard deviation (coefficient of variation)

^b^Edi min = minimum electrical activity of the diaphragm

^c^Edi peak = peak electrical activity of the diaphragm

^d^Edi swing = peak – minimum electrical activity of the diaphragm

Edi minimum and Edi peak were compared during various states (Table 3). There were no differences observed with feeding (pre- vs post-prandial). Edi minimum was significantly higher when infants were awake (4.07 [±1.45] μV vs 2.69 [±0.82] μV; *p* < 0.001). Similarly, Edi peak was significantly higher when infants were awake (13.43 [±4.49] μV vs 9.11 [±2.92] μV; *p* < 0.001]. During skin-to-skin care, Edi peaks were significantly lower (7.01 [±0.50] μV vs 10.14 [±3.53] μV; *p* = 0.013) and less variable (*p* = 0.04).

**Table 3:** Edi Min, Edi Peak, and Respiratory Rate during the various studied states^a,b,c^

	N	Edi Min (μV)	Edi Peak (μV)	Respiratory Rate
Feeding states				
Pre-prandial	23	3.01 ± 1.14 (CV 0.38)	9.95 ± 3.82 (CV 0.38)	48.57 ± 7.21
Post-prandial	23	3.34 ± 1.15 (CV 0.34)	10.89 ± 3.82 (CV 0.35)	48.36 ± 6.16
*p*-value		0.171	0.172	0.883
Resting states				
Asleep	24	2.69 ± 0.82 (CV 0.30)	9.11 ± 2.92 (CV 0.32)	47.37 ± 5.69
Awake	24	4.07 ± 1.45 (CV 0.36)	13.43 ± 4.49 (CV 0.33)	49.28 ± 6.64
*p*-value		<0.001	<0.001	0.093
Kangaroo Care				
No skin-to-skin care	24	3.04 ± 0.95 (CV 0.31)	10.14 ± 3.53 (CV 0.35)	47.67 ± 5.40
Skin-to-skin care	3	2.51 ± 0.32 (CV 0.13)	7.01 ± 0.50 (CV 0.07)	55.20 ± 5.79
*p*-value		0.099	0.013	0.198

^a^Data is presented as mean ± standard deviation (coefficient of variation)

^b^Edi min = minimum electrical activity of the diaphragm

^c^Edi peak = peak electrical activity of the diaphragm

Differences between Edi minimums did not reach statistical significance. There were no significant differences between male and female infants.

## Discussion

The observational data in this study represent Edi waveforms from a large cohort of healthy preterm and term newborn infants, which we have used to define reference values for Edi minimum (1–5 μV) and Edi peak (3–17 μV). These values can be used as a reference in clinical practice to define a goal for electrical activity in infants requiring respiratory support. This is particularly relevant in infants being supported with NAVA as breathing support is always proportional to the infant’s Edi activity (specifically, proportional to the difference between the Edi peak and Edi minimum). There was no significant difference found between the preterm and term population in the study suggesting that diaphragm electrical activity is similar in infants that are spontaneously breathing without support. This supports the use of these reference values in both preterm and term infants.

We noted that diaphragm electrical activity was significantly higher when infants were awake. This is an expected finding as the sleep stage is characterised by slower, more regular respiration rates [10]. It is also consistent with previous studies, which demonstrated that peak Edi activity was 60% higher in the awake state [3]. Peak Edi was also significantly lower during skin-to-skin care, which has previously been reported and is not surprising given the known positive effects of skin-skin care on cardiorespiratory stability and sleep quality [11–13]. It should be noted, however, that only three infants had skin-to-skin care during their respective observation periods.

We found no significant difference in Edi waveforms when comparing waveforms before and after feeds. This is consistent with previous studies investigating changes in Edi with naso/orogastric feeds [14]. There are other studies that have shown a post-prandial decrease in peak Edi, however, this was in a small population of term infants who were suck feeding [3]. The majority of infants in this study were predominantly fed with a naso/orogastric tube, which is likely to explain the discrepancy.

It is important to recognise that the Edi waveform is variable in individual infants. Infants often have irregular, periodic breathing patterns that can fluctuate with time. The effects of clinical variables such as sigh breaths, apnoeas, use of accessory muscles and changing respiratory rate are not reflected when the Edi waveform is averaged. When using reference ranges, the trend in an individual infant’s Edi waveform is more important than any single value. There is also variability between infants, which explains why there is an overlap between the reference ranges for Edi minimum and Edi peak (it is not possible for the Edi minimum to be greater than the Edi peak at any one point in time).

There were some important limitations to acknowledge with respect to this study. Although these data represent the largest reported cohort of spontaneously breathing preterm and term infants, the sample size does not allow for a true reference range based on gestation. We found that the electrical diaphragm activity is similar in preterm infants and term infants, but these were all infants who did not require respiratory support. This reflects an increasingly small percentage of the population as gestation decreases. Consequently, we were not able to recruit infants less than 29 weeks gestation where differences in diaphragm activity may be more apparent. We also acknowledge that the inclusion of infants admitted to a special care nursery may not represent a true healthy newborn population. Although infants were subjectively breathing comfortably at the time of enrolment, some residual subclinical respiratory disease cannot be ruled out. We are reassured that our findings are similar to previous published data [3, 5, 6]. Finally, it is important to note that Edi signals may be affected by equipment and software factors, such as electrode configuration or signal amplification. This again highlights the value of trend monitoring when interpreting Edi data in an individual infant.

## Conclusions

In summary, reference Edi minimum and peak values were established for both preterm and term neonates. These values can be used as a guide for clinicians when using diaphragm-triggered modes on respiratory support in newborn infants to ensure an optimal level of respiratory support.

## Supplementary Information

**Additional file 1.**

**Additional file 2.**

## Data Availability

The datasets used and/or analysed during the current study are available from the corresponding author on reasonable request.

## Abbreviations

Edi: Electrical activity of the diaphragm
SD: Standard deviation
NAVA: Neurally adjusted ventilatory assist
NICU: Neonatal Intensive Care Unit
