# Supplementary material for: Reference values for diaphragm electrical activity (Edi) in newborn infants
Source: BMC Pediatr. 2022 Sep 23;22:559. doi: 10.1186/s12887-022-03619-1 (PMC9502911; doi:10.1186/s12887-022-03619-1)
Supplement: Supplementary file 1 — Additional file 1. [file 12887_2022_3619_MOESM1_ESM.docx]

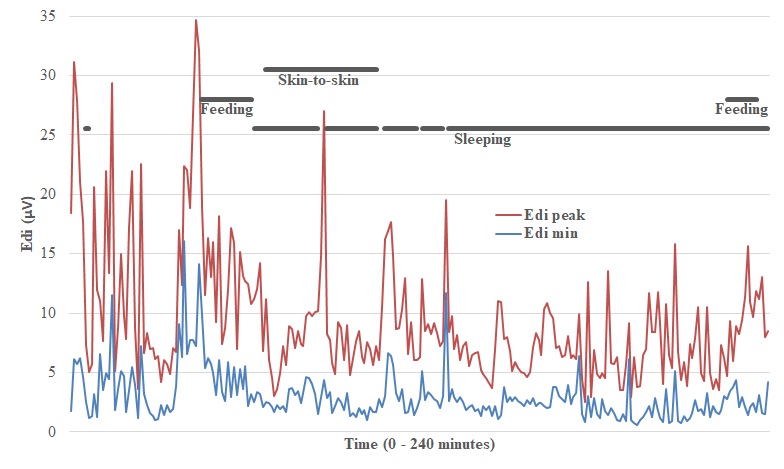


Appendix 1: Edi peak (red) and Edi min (blue) values plotted across a 240-minute period, with annotations marking the various states of skin-to-skin contact, feeding, and sleeping for a selected neonate.
